# Metabolic Footprint of *Treponema phagedenis* and *Treponema pedis* Reveals Potential Interaction Towards Community Succession and Pathogenesis in Bovine Digital Dermatitis

**DOI:** 10.3390/pathogens13090796

**Published:** 2024-09-14

**Authors:** Hector M. Espiritu, Edeneil Jerome P. Valete, Lovelia L. Mamuad, Myunghwan Jung, Man-Jeong Paik, Sang-Suk Lee, Yong-Il Cho

**Affiliations:** 1 Department of Animal Science and Technology, Sunchon National University, Suncheon-si 57922, Jeollanam-do, Republic of Korea; hectorme@scnu.ac.kr (H.M.E.); 1225054@s.scnu.ac.kr (E.J.P.V.); loveliamamuad2306@gmail.com (L.L.M.); rumen@scnu.ac.kr (S.-S.L.); 2 Department of Microbiology, College of Medicine, Gyeongsang National University, Jinju 52727, Gyeongsangnam-do, Republic of Korea; mjung@gnu.ac.kr; 3 College of Pharmacy, Sunchon National University, Suncheon-si 57922, Jeollanam-do, Republic of Korea; paik815@scnu.ac.kr

**Keywords:** bovine digital dermatitis, *Treponema*, exometabolomics, polymicrobial disease

## Abstract

Bovine digital dermatitis (BDD) is a cattle infection causing hoof lesions and lameness, with treponemes as key pathogens. We analyzed the metabolic activity of *Treponema phagedenis* and *Treponema pedis* using gas chromatography–mass spectrometry for organic acids (OAs), amino acids (AAs), and fatty acids (FAs), and high-performance liquid chromatography for short-chain fatty acids (SCFAs). Key findings include a 61.5% reduction in pyruvic acid in *T. pedis* and 81.0% in *T. phagedenis*. 2-hydroxybutyric acid increased by 493.8% in *T. pedis*, while succinic acid increased by 31.3%, potentially supporting *T. phagedenis*. Among AAs, glycine was reduced by 97.4% in *T. pedis* but increased by 64.1% in *T. phagedenis*. Proline increased by 76.6% in *T. pedis* but decreased by 13.6% in *T. phagedenis*. Methionine and glutamic acid were competitively utilized, with methionine reduced by 41.8% in *T. pedis* and 11.9% in *T. phagedenis*. Both species showed significant utilization of palmitic acid (reduced by 82.8% in *T. pedis* and 87.2% in *T. phagedenis*). Butyric acid production increased by 620.2% in *T. phagedenis*, and propionic acid increased by 932.8% in *T. pedis* and 395.6% in *T. phagedenis*. These reveal metabolic interactions between the pathogens, contributing to disease progression and offering insights to BDD pathogenesis.

## 1. Introduction

Many microorganisms live in complex polymicrobial biofilm communities, as single-species planktonic microbes are not common [1]. These communities are composed of different species with specialized phenotypes performing distinct functions, and several species within the community can work together to carry out more complex processes. Some of these interactions can range from intense competition for nutrients and niches to highly evolved cooperative mechanisms that support cooperative growth between multiple species [2]. Such complex interactions between members of the polymicrobial biofilm may play a pivotal role in the pathogenesis of most known infections [3]. A growing awareness of these interactions, as well as a desire to better understand the processes governing them, has led to a transition from monomicrobial to polymicrobial biofilm studies in various disease models [4].

One of the major diseases that exhibits polybacterial etiology is bovine digital dermatitis (BDD). BDD is a major problem in the livestock and dairy sector because it reduces [both welfare and economic efficiency of animals globally. It is a cutaneous infection characterized by an ulcerative and proliferative lesion on the hoof that affects the weight-bearing distribution, gait, and posture of affected animals and often leads to lameness. Previous investigations have reported that this disease has a complex microbiome due to the dysbiosis caused by the overabundance of opportunistic pathogens which mainly include multiple *Treponema* species [5,6,7,8,9]. Despite five decades since its initial report, limited progress has been made in the study of the fundamental mechanisms underlying BDD pathogenesis. This is primarily due to the fastidious behavior exhibited by the anaerobic group of bacteria involved. Currently, four *Treponema* species have been successfully recovered from BDD lesions, three of which are considered as the main pathogens involved because of their high abundance level persistently found in most lesions, namely *T. phagedenis*, *T. pedis*, and *T. medium* [10,11,12,13,14]. The persistence of these treponemes in lesions has led to a series of investigations of their properties to decipher their role in the pathogenesis of BDD [7,15,16].

In the polybacterial consortium within BDD, the interactions among members of the community are undeniably complex. Competition for intermediates or products, or cooperative interactions, such as the beneficial metabolic exchange known as “cross-feeding”, may be at play [2]. Identification of the interactions among the bacteria responsible for BDD may offer important clues about its etiology. Exometabolomics, or metabolic footprinting, the study of small metabolite profiles in extracellular environments, is a method that is ideally suited for this. Insights into the metabolic activities of certain microbial strains or communities can be obtained by analyzing metabolites absorbed from or released into culture medium as individual bacterial strain preferences can be characterized to identify the likelihood of competing with other strains [1]. In this study, we aimed to elucidate the metabolic footprint profiles of two *Treponema* species, *T. phagedenis* and *T. pedis*, isolated from BDD in Korean dairy cattle. By analyzing their metabolic footprints in monocultures, we aimed to compare key metabolites, namely organic acids (OAs), amino acids (AAs), and fatty acids (FAs), measured using targeted techniques like gas chromatography–tandem mass spectrometry (GC-MS/MS) and high-performance liquid chromatography (HPLC). OAs are involved in energy production and basic metabolic processes, AAs are essential for protein synthesis and bacterial growth, and FAs are important for cell membranes and energy storage. This will provide us with clues about both the potential metabolic interactions between *T. phagedenis* and *T. pedis* and how these metabolites might affect the host tissues.

## 2. Materials and Methods

### 2.1. Isolates and Culture Medium

Bacterial cultures of *T. phagedenis* HNW1 [17] and *T. pedis* GNW45 [18] isolated previously were prepared using an optimized *Treponema* enrichment broth (OTEB, Anaerobe Systems, Morgan Hill, CA, USA) supplemented with 10% fetal bovine serum to provide necessary nutrients for anaerobic growth [17]. Each culture was prepared in triplicate, alongside a negative control consisting of the same OTEB medium with 10% FBS but without bacterial inoculum. This control was used to establish baseline levels of metabolites present in the medium, ensuring that any observed metabolic changes in the experimental cultures were attributable to bacterial activity. Post-incubation, all cultures, including the negative control, were examined microscopically for any bacterial growth or contamination. Following this, cultures were centrifuged at 4000 rpm for 10 min to harvest the supernatants. The supernatants were then sterile-filtered using a 0.2 µm filter to remove any remaining cells and stored at −80 °C for subsequent analysis.

### 2.2. Metabolomic Profiling

The quantification of OAs, AAs, and FAs in the culture supernatants was performed using GC-MS/MS which followed the methodology described by Kim et al. (2020) [19]. To prepare the samples for GC-MS/MS analysis, a derivatization protocol was employed to convert the metabolites into volatile and stable derivatives. The derivatization involved three key reactions: ethoxycarbonylation (EOC) to modify amine groups in AAs, methoximation (MO) to protect carbonyl groups, and tert-butyldimethylsilylation (TBDMS) to derivatize hydroxyl and carboxyl groups. These reactions were essential for converting the metabolites into forms suitable for GC-MS/MS analysis for precise detection and quantification. The GC-MS/MS analysis was conducted on a Shimadzu TQ 8040 triple quadrupole mass spectrometer (Kyoto, Japan) with an Ultra-2 capillary column. The instrument operated in split-injection mode with helium as the carrier gas and argon as the collision gas. Specific chromatographic conditions were applied to optimize the separation and detection of each metabolite class: For OAs, the GC oven temperature was initially set at 100 °C for 2 min, then ramped to 250 °C at 10 °C/min, followed by an increase to 300 °C at 20 °C/min. AAs were analyzed by maintaining the oven temperature at 140 °C for 3 min before increasing to 300 °C at 8 °C/min. FAs were measured by holding the temperature at 100 °C for 3 min, followed by a rise to 200 °C at 20 °C/min, then 260 °C at 1.5 °C/min, and finally 300 °C at 20 °C/min. The MS employed electron impact ionization at 70 eV and multiple reaction monitoring (MRM) mode, focusing on specific precursor and product ion transitions for accurate metabolite identification. Calibration curves were constructed using standards to ensure accurate quantification of metabolite concentrations in the samples.

Additional analysis for the determination of the concentration of short-chain fatty acids (SCFAs) was conducted using high-performance liquid chromatography (HPLC). The analysis utilized an Agilent 1200 Series HPLC System with a UV detector set at 210 and 220 nm. Samples were eluted isocratically with 0.0085 N H_2_SO_4_ at a flow rate of 0.6 mL/min and a column temperature of 35 °C, following a previous protocol [17].

### 2.3. Data Analysis

The data obtained from GC-MS/MS and HPLC analyses were subjected to statistical evaluation. Analysis of variance (ANOVA) followed by Duncan’s multiple range test were used to assess significant differences between metabolite concentrations in the different *Treponema* species. All statistical analyses were performed using SAS version 9.4. The results were normalized against baseline values obtained from the control using relative normalization. These normalized values were then visualized using radar plots created in Microsoft Excel (https://www.microsoft.com/en-au/microsoft-365/excel, accessed on 15 Ausgust 2024) to illustrate patterns of metabolite utilization and production.

## 3. Results

A total of 40 metabolites, which included 11 OAs, 19 AAs, six FAs, and five SCFAs, were analyzed. Out of these, a total of eight OAs, nine AAs, four FAs, and all SCFAs have significant differences based on the concentration between the isolates and the control.

### 3.1. Organic Acids (OAs)

**Figure 1:**
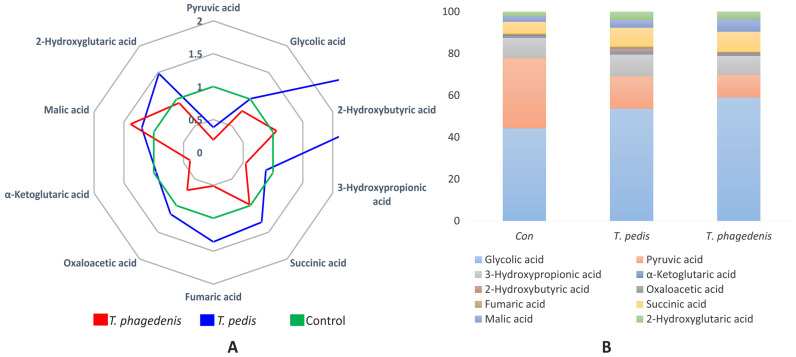
(**A**) Illustrates the normalized concentration values of OAs in the culture medium negative control (Con), *T. pedis*, and *T. phagedenis*, with each axis representing a different OA; (**B**) shows the percentage composition of each OA in the culture media. Each segment represents a different OA, indicating their relative abundance.

The concentrations of OAs quantified in the culture medium are detailed in Table 1. Significant differences were observed for several OAs, including pyruvic acid, glycolic acid, 2-hydroxybutyric acid (2-HBA), 3-hydroxypropionic acid (3-HPA), succinic acid, fumaric acid, oxaloacetic acid, and α-ketoglutaric acid. Both *T. pedis* and *T. phagedenis* exhibited significant reductions in pyruvic acid levels, with decreases of 61.5% and 81.0%, respectively, compared to the control, suggesting competition for this metabolite. Glycolic acid levels were reduced by 21.8% in *T. phagedenis* but remained stable in *T. pedis*. There was a notable increase of 493.8% in 2-HBA in *T. pedis*, while *T. phagedenis* remained unchanged. 3-HPA levels decreased by 11.9% in *T. pedis* and 45.7% in *T. phagedenis*. Succinic acid levels increased by 31.3% in *T. pedis* but showed no significant change in *T. phagedenis*. Fumaric acid utilization increased by 36.7% in *T. pedis* and decreased by 48.4% in *T. phagedenis*, indicating possible cooperative interactions. Oxaloacetic acid increased slightly by 15.8% in *T. pedis* but decreased by 29.2% in *T. phagedenis*. α-Ketoglutaric acid decreased by 60.7% in *T. phagedenis* but remained stable in *T. pedis*. The normalized concentrations of these metabolites are depicted in Figure 1A through a radar plot, which illustrates whether each metabolite was utilized or produced by the bacteria. Figure 1B presents the percentage composition of each OA, offering a comparative view of their relative abundances across different conditions.

### 3.2. Amino Acids (AAs)

The concentration of AAs in the culture medium varied significantly, particularly for alanine, glycine, proline, methionine, serine, threonine, aspartic acid, glutamic acid, and glutamine, as detailed in Table 2. *T. pedis* showed a 40.9% increase in alanine levels compared to the control, while *T. phagedenis* exhibited a 20.8% increase. A significant reduction in glycine was observed for *T. pedis*, which showed a 97.4% decrease compared to the control, whereas *T. phagedenis* exhibited increased glycine levels, with a 64.1% increase. Proline concentrations were significantly higher in *T. pedis*, showing a 76.6% increase compared to the control, while *T. phagedenis* exhibited a 13.6% reduction. Significant reductions in methionine were noted across both bacterial species, with *T. pedis* showing a 41.8% decrease and *T. phagedenis* showing a 11.9% decrease compared to the control.

Threonine levels increased by 58.8% in *T. pedis* compared to the control, but *T. phagedenis* showed a 45.0% reduction. Aspartic acid levels decreased by 26.8% in *T. pedis*, while *T. phagedenis* exhibited a 49.4% increase compared to the control. Glutamic acid was not detected in *T. phagedenis*, indicating substantial utilization, while *T. pedis* showed a reduction of 28.2%. Glutamine levels were significantly reduced in *T. phagedenis* (100% utilization), and *T. pedis* showed a 19.2% decrease compared to the control.

This pattern of AA metabolism suggests potential metabolic cross-feeding between the treponemes for amino acids like proline and threonine, and metabolic competition for methionine, glutamic acid, and glutamine. Serine appears to be commensally shared between the two species, as it showed only a slight reduction in both.

**Figure 2:**
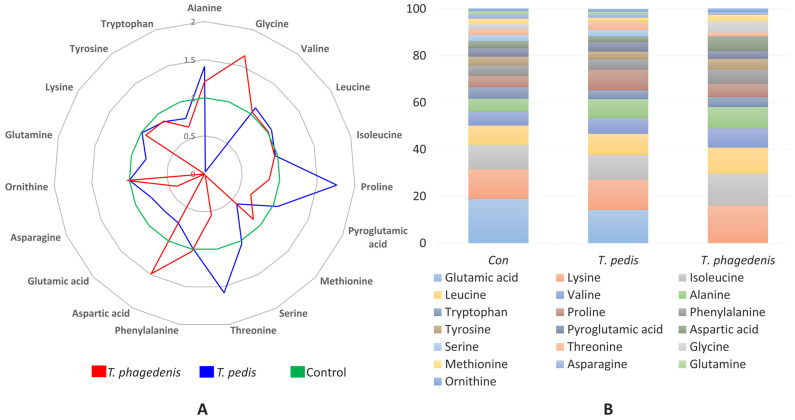
(**A**) Illustrates the normalized concentration values of AAs in the culture medium negative control (Con), *T. pedis*, and *T. phagedenis*, with each axis representing a different AA; (**B**) shows the percentage composition of each AA in the culture media. Each segment represents a different AA, indicating their relative abundance.

The normalized concentrations of these metabolites are depicted in Figure 2A through a radar plot, which illustrates whether each amino acid was utilized or produced by the bacteria. Figure 2B presents the percentage composition of each AA, offering a comparative view of their relative abundances across different conditions.

### 3.3. Fatty Acids (FAs/SCFAs)

The *Treponema* species exhibited significant utilization of FAs, particularly cis-9-Hexadecenoic acid, palmitic acid, linoleic acid, and oleic acid, as evidenced by their reduced levels in both *T. pedis* and *T. phagedenis* compared to the control, as detailed in Table 3. Cis-9-Hexadecenoic acid levels were significantly reduced by about 80.4% in *T. pedis* and 87.3% in *T. phagedenis* compared to the control. Palmitic acid levels showed an 82.8% reduction in *T. pedis* and an 87.3% reduction in *T. phagedenis*. Linoleic acid levels decreased by 86.3% in *T. pedis* and 47.8% in *T. phagedenis*. Oleic acid levels were lower by 42.1% in *T. pedis* and 48.2% in *T. phagedenis*. Although octadecanoic acid was also utilized by both spirochetes, the changes were not statistically significant.

Distinct patterns of production and utilization were observed for metabolites measured through HPLC, including SCFAs and lactic acid, as shown in Table 3. Notably, there was a significant increase in butyric acid production, particularly in *T. phagedenis*, which exhibited a dramatic 620.1% increase, while *T. pedis* showed a 317.1% increase compared to the control, highlighting butyric acid as a key metabolite in BDD. Propionic acid production also showed striking differences. *T. pedis* exhibited a remarkable 932.8% increase compared to the control, while *T. phagedenis* showed a 395.6% increase. This suggests significant metabolic activity in both species due to higher levels of propionic acid produced. Formic acid production was exclusively observed in *T. phagedenis*, not detected in the control or *T. pedis*. In contrast, *T. pedis* exhibited higher production of acetic acid (130.8% increase) compared to *T. phagedenis*, which showed a lower increase of 20.5%. Lactic acid, though not an SCFA but are commonly produced by SCFA-producing bacteria, shows that its concentrations were significantly reduced in both *T. pedis* and *T. phagedenis*, with a reduction of 41.5% and 50.2%, respectively, indicating substantial utilization by these species.

**Figure 3:**
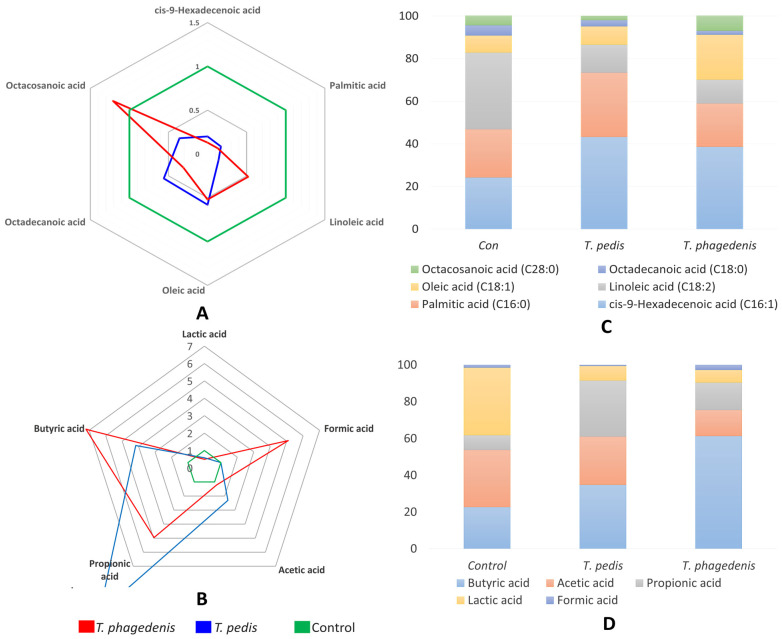
The radar plots show normalized concentration values of FAs (**A**) and SCFAs (**B**) between negative control (Con), *T. pedis*, and *T. phagedenis* in the culture medium. The column charts depict the percentage composition of each FA (**C**) and SCFA (**D**) in the culture media. Each segment represents a metabolite presenting their relative abundance.

The normalized concentrations of these metabolites are depicted in Figure 3A,C through a radar plot, which illustrates whether each fatty acid or SCFA was utilized or produced by the bacteria. Figure 3B,D presents the percentage composition of each FA and SCFA, offering a comparative view of their relative abundances across different conditions.

## 4. Discussion

In this study, we used exometabolomics, also known as metabolic footprinting, to examine how two *Treponema* species change small molecules in their environment by comparing culture media with and without the bacteria. This method examines how cells modify these molecules in their niche [20], helping us understand how these bacteria interact in the BDD bacterial community. By analyzing AAs, FAs, and OAs, we can see how the bacteria either cooperate or compete for resources. Our results showed that both *Treponema* species have unique metabolic patterns, using up many essential AAs and OAs while consuming key FAs, suggesting they have adapted to survive and thrive in their environment. The observed differences in metabolic profiles between *T. phagedenis* and *T. pedis* are expected given their distinct species identities, but these differences provide biologically significant insights into how each species adapts to and potentially influences the BDD microenvironment. For example, the production of butyric acid by *T. phagedenis* might contribute to its survival or affect the host’s immune response differently than *T. pedis*. These variations could play a role in how these bacteria interact with the host and with other microorganisms in the community, potentially impacting disease progression. It is also important to acknowledge certain limitations; for instance, the use of FBS as a nutrient source, though obtained from the same batch across all experiments, presents a level of complexity that may not fully replicate the natural environment within a bovine host. In vivo, *Treponema* species would interact with a range of host tissues, immune responses, and other members of the polymicrobial community, factors that our in vitro conditions cannot entirely mimic. This means that while our findings are robust within the context of the experimental setup, caution should be exercised when extrapolating these results to natural, in vivo conditions. Furthermore, BDD is known to be a polymicrobial infection, and our focus on monoculture analyses in this study was aimed at establishing baseline metabolic activities and understanding the differences between *T. phagedenis* and *T. pedis*. However, the complexity of BDD urges that future research would benefit from exploring mixed cultures, not only among *Treponema* spp. but also including other non-treponeme bacteria commonly found in lesions, such as *Fusobacterium* spp., *Porphyromonas* spp., and *Dichelobacter* spp. [7]. Analyzing a mixed metabolic profile could provide deeper insights into potential metabolic co-dependencies and interactions among these diverse microbial species. Such studies could reveal whether these bacteria exhibit metabolic cooperation or competition when coexisting within the same environment, offering a more comprehensive understanding of their roles within the BDD microbial community.

According to Freilich et al. [21], metabolic cooperation occurs when one bacterium produces a metabolite that another uses, while competition happens when both use the same metabolite. Our findings indicate both cooperative and competitive interactions between the species, providing insight into their roles in the BDD bacterial community. These results enhance our understanding of *Treponema* biology and suggest possible targets for treatments to disrupt these bacteria’s metabolic pathways and control disease progression.

### 4.1. Organic Acids

For the OAs presented in Table 1 and Figure 1, pyruvic acid is a key metabolic requirement for both *T. pedis* and *T. phagedenis*, as evidenced by their competitive interaction for this metabolite. Previous studies have established pyruvic acid as a crucial intermediate in bacterial carbohydrate metabolism [22]. As a monocarboxylic acid produced at the end of glycolysis, pyruvic acid can serve as the sole carbon source for some bacteria, such as *E. coli* [23]. *Treponema* species have also been documented to be pyruvate-dependent. For example, Nichols and Baseman demonstrated that the virulent *T. pallidum* selectively uses pyruvate as a carbon source, producing CO_2_ and acetate as degradation end products [24]. Additionally, the glutathione metabolism of *T. denticola* rapidly consumes pyruvate, enhancing bacterial colonization and producing volatile sulfur compounds (VSCs) like hydrogen sulfide (H_2_S), which plays a significant role in pathogenic changes in human periodontal tissues through hemoxidation and hemolysis [25]. The production of VSCs such as H_2_S and methyl mercaptan as metabolic byproducts could explain the strong malodor associated with BDD, as these compounds are highly toxic and contribute to host tissue damage.

Regarding glycolic acid, no interaction was observed between the two *Treponema* species. The significant reduction of glycolic acid by *T. phagedenis* suggests that this bacterium might utilize it as a carbon and energy source, potentially enhancing its colonization ability. A similar mechanism has been observed in other colonizing bacteria, such as *Mycoplasma pneumoniae*, which uses glycerol [26]—modulated by glycolic acid [27]—as a carbon and energy source, implicating its role in virulence.

Among the OAs, we observed an overproduction of 2-hydroxybutyric acid by *T. pedis*. Although limited information is available regarding this OA’s role in bacterial virulence in host tissues, it is related to butyrate, which has been shown to have both positive and negative effects depending on tissue localization. We also observed that 3-hydroxypropionic acid levels were lower, indicating competitive utilization between the treponemes. This suggests that 3-hydroxypropionic acid may play a pivotal role in bacterial community succession, potentially sourced from other propiogenic bacteria in BDD or from the host itself.

Succinic acid was uniquely produced by *T. pedis*. Similar to butyric acid, elevated levels of succinic acid have been associated with periodontitis, promoting dysbiosis, inflammation, and bone loss [28]. Historically, succinate produced by *Bacteroides* spp. in mixed intra-abdominal infections has been shown to impair host polymorphonuclear leukocyte function [29]. In periodontal disease, *Tannerella forsythia*, a member of the “red complex”, can produce succinate by reducing fumaric acid, thereby promoting the growth of other “red complex” bacteria, such as *P. gingivalis* [30]. A similar mechanism could be occurring in BDD, as we observed that both succinic acid and fumaric acid were produced by *T. pedis*, potentially promoting the growth of *T. phagedenis*.

For oxaloacetic acid and alpha-ketoglutaric acid, there is limited information on their involvement in tissue damage and virulence. However, oxaloacetic acid has been reported to support the anaerobic growth of *Actinobacillus pleuropneumoniae*, a porcine lung pathogen [31], while alpha-ketoglutaric acid is known to assimilate nitrogen in diverse bacterial communities in the gut [32]. This suggests that these metabolites may be more involved in cooperative metabolism for microbiome succession in BDD.

### 4.2. Amino Acids

Pathogens require AAs to support their physiological functions, and changes in the availability of AAs can significantly affect pathogen growth and expression of virulence factors [32]. In this study, we observed several AAs that are cooperatively regulated between *T. pedis* and *T. phagedenis*, as presented in Table 2 and in Figure 2. Alanine is crucial for bacterial growth and viability [33], and studies have shown that alanine levels are altered at infection sites, such as in periodontitis, where its levels are higher in patients before treatment [34,35].

Aspartic acid showed cooperative interaction between the two treponemes, suggesting its role in their survival within the microbiome. Szafranski et al. (2015) noted increased aspartate degradation in chronic periodontitis patients [36]. Glutamic acid and glutamine were undetectable in *T. phagedenis*, indicating complete utilization. Closely related treponemes, like the Reiter strain of *T. pallidum*, are known to degrade AAs such as arginine, histidine, serine, threonine, and glutamic acid [37]. *T. phagedenis* is closely related to *T. pallidum*, having been previously classified as one of the non-pathogenic strains of *T. pallidum* [38]. Although evidence of glutamine’s role as a virulence factor in treponemes is limited, it has been shown that oral supplementation of glutamine in rats reduces periodontitis progression [39]. The absence of glutamine at infection sites could be due to the utilization of the pathogen from the host, potentially causing tissue damage or toxic byproducts from glutamine metabolism. Glutamate levels were also lower in both treponemes. In chronic periodontitis patients, reduced glutamate is linked to its consumption by pathogens like *P. gingivalis* and *F. nucleatum* [40]. Glutamic acid is involved in vitamin B12 and porphyrin biosynthesis, promoting proinflammatory responses in skin diseases such as acne vulgaris [41]. Pyroglutamic acid, although utilized in modest levels by *T. phagedenis* and moderately produced by *T. pedis*, study suggests cooperation, and its excretion in periodontitis suggests disruptions in oxidative stress response and gluta-thione synthesis [42]. Similarly, *T. denticola* can convert glycine into serine using serine hydroxymethyltransferase, and further deaminates serine to produce pyruvate, which is metabolized into acetate or lactate [43].

Threonine production by *T. pedis* may be utilized by *T. phagedenis*. This interaction parallels those in periodontitis, where pathogens like *P. gingivalis* assimilate serine and threonine from culture media [44]. Threonine can also be synthesized from glycine or homoserine, as seen in *Salmonella* Typhimurium, aiding intra-host survival [45]. Proline utilization promotes biofilm growth, suggesting the cooperative regulation of proline by these treponemes enhances biofilm formation and colonization of BDD pathogens [46].

The significant reduction of methionine in the spirochetes might be due to the action of L-methionine-α-deamino-γ-mercaptomethane lyase (METase), which breaks down L-methionine into α-ketobutyrate, ammonia, and methyl mercaptan—another VSC [47]. This has been observed in human periodontitis with pathogens like *P. gingivalis* [48] and *T. denticola* [47] after supplementation with heat-inactivated fetal bovine serum. The absence of asparagine in the substrate reduces the formation rate of round bodies in *T. denticola*, highlighting its importance in forming bacterial persisters during stress or starvation [49].

### 4.3. Fatty Acids

The significant utilization of FAs by *Treponema* species has been supported by studies from the 1970s and 1980s on pathogenic treponemes, such as *T. pallidum* and *T. denticola* [50]; however, there has been less research on other *Treponema* species. Our study highlights that FAs are essential metabolic sources for *T. pedis* and *T. phagedenis*, with competitive interactions observed between them. FAs are crucial for biosynthesis of cellular membranes and serve as an energy source through catabolism [51]. *Treponema* species rely on FAs, which constitute a significant portion of their cell mass, primarily as phospholipids in their membranes that enclose the cell body and periplasmic space with peptidoglycan and endoflagellae [52]. Although there is limited evidence specific to BDD treponemes, competition within the microbiome may drive these bacteria to utilize host-derived FAs, similar to other pathogenic bacteria [53]. Previous studies have linked palmitic acid to periodontitis pathogenesis due to its ability to increase pro-inflammatory cytokines [54]. Other reports indicate that host-derived fatty acids serve as nutrient sources for pathogens, such as in *Mycobacteria* [55].

There are currently limited reports of SCFA production or utilization linked with *T. pedis* [56]. On the other hand, *T. phagedenis* has been reported to produce SCFAs and alcohol as fermentation end products [50]. Additionally, the *T. phagedenis* Reiter strain has been known to produce acetic and N-butyric acids as major fermentation end products, while propionic acid is less common [57]. In vitro trials involving *Cutibacterium acnes* (formerly *Propionibacterium acnes*), the causative agent of inflammatory skin disease acne, have shown that strains secrete propionic acid, which contributes to their pathogenicity by affecting keratinocyte cell growth and causing cytotoxicity in a strain-specific and dose-dependent manner [58]. Significant production of propionic acid could explain the glutamic acid utilization, as well as the hyperinflammatory lesions of BDD. In addition, a previous report found that *T. phagedenis* isolated from BDD produced formic, acetic, and butyric acids, although quantitative data were not provided [59]. The quantitative results for SCFAs obtained in this study are consistent with these earlier findings.

It is assumed that BDD forms through successive waves of bacterial colonization, where early colonizers create an environment favorable for later anaerobic colonizers. Previously, pathogenic treponemes were thought to colonize later once anaerobic conditions were established and mid-colonizers altered nutrient availability by providing SCFAs. However, our results suggest that treponemes themselves produce most SCFAs, particularly butyric acid and propionic acid. Numerous studies have explored butyrate’s role in periodontitis progression [60,61,62,63]. For example, Shirasugi et al. (2018) concluded that elevated butyrate levels are associated with increased periodontitis progression [60]. Although butyrate has beneficial functions in the gut and nervous system, it has detrimental effects in the oral cavity [63], which may be similar in BDD. Formic acid was observed to promote tumor progression by *Fusobacterium nucleatum* [64].

Treponemes are frequently detected in deeper parts of BDD lesions, where they utilize host-derived AAs and FAs, resulting in the production of SCFAs that may cause dysbiosis and host tissue damage. Recent studies on periodontitis have shown increased SCFA levels as the disease progresses [60,65]. Our findings suggest that BDD treponemes utilize medium- to long-chain FAs, producing polyamines like cadaverine and putrescine. SCFAs are produced from AA and carbohydrate metabolism, creating an acidic microenvironment favorable for treponemal growth and potentially triggering host pro-inflammatory immune responses.

## 5. Conclusions

This study represents the first detailed exploration of the metabolic footprint of *T. phagedenis* and *T. pedis*, two key pathogens associated with BDD. The results revealed significant interactions between these species, including both metabolic competition and cross-feeding. OAs like pyruvic acid and fumaric acid were competitively consumed, while 2-HBA and succinic acid were produced cooperatively by *T. pedis*, potentially supporting the growth of *T. phagedenis*. Among AAs, glycine, proline, and threonine were regulated cooperatively, while methionine and glutamic acid were competitively utilized, highlighting important metabolic dependencies that may affect bacterial persistence within BDD lesions. Both species demonstrated significant utilization of FAs such as palmitic acid and linoleic acid, which are essential for their survival and virulence. SCFAs were also central to their metabolic strategies, with *T. phagedenis* showing increased butyric acid production and both species exhibiting high levels of propionic acid, contributing to the damaging environment of BDD lesions. These insights not only enhance our understanding of the complex polybacterial community within BDD but also highlight potential metabolic targets for therapeutic intervention. Further research is necessary to explore these metabolic interactions in more complex polymicrobial settings that mimic the in vivo environment more closely.

## Figures and Tables

**Table 1:** Quantitative analysis of organic acid profiles in culture media of *Treponema* spp.

OA (ng/5 µL)	Control	*T. pedis*	*T. phagedenis*	SEM	*p*-Value
Pyruvic acid	1258.4 ^a^	484.2 ^b^	239.6 ^b^	64.76	<0.001
Glycolic acid	1666.9 ^a^	1680.9 ^a^	1302.5 ^b^	48.0	<0.001
2-Hydroxybutyric acid	8.0 ^b^	47.5 ^a^	8.5 ^b^	0.69	<0.001
3-Hydroxypropionic acid	365.8 ^a^	322.3 ^a^	198.5 ^b^	28.69	0.019
Succinic acid	217 ^b^	284.9 ^a^	213.1 ^b^	8.74	<0.001
Fumaric acid	12 ^b^	16.4 ^a^	6.2 ^c^	0.77	<0.001
Oxaloacetic acid	17.1 ^a^	19.8 ^a^	12.1 ^b^	0.85	<0.001
α-Ketoglutaric acid	34.1 ^a^	32.8 ^a^	13.4 ^b^	1.56	<0.001
Malic acid	95.6	114.5	132.7	10.89	0.205
2-Hydroxyglutaric acid	82.2	121.6	76.3	15.45	0.231

Different superscripts ^(a,b,c)^ within the same row indicate statistically significant differences between groups (control, *T. pedis*, *T. phagedenis*) based on one-way ANOVA and Duncan’s multiple range test (DMRT) (*p* < 0.05).

**Table 2:** Quantitative analysis of amino acid profiles in culture media of *Treponema* spp.

OA (ng/2 µL)	Control	*T. pedis*	*T. phagedenis*	SEM	*p*-Value
Alanine	59.1 ^c^	83.3 ^a^	71.4 ^b^	52.09	<0.001
Glycine	23.4 ^b^	0.6 ^c^	38.4 ^a^	13.79	<0.001
Valine	62.3	68.7	64.4	46.39	0.556
Leucine	86.9	92.4	87.3	60.69	0.188
Isoleucine	112.5	109.4	107.8	77.06	0.933
Proline	50.8 ^b^	89.7 ^a^	43.9 ^b^	46.78	<0.001
Pyroglutamic acid	39.2	41.7	26.3	24.73	0.060
Methionine	24.4 ^a^	14.2 ^b^	21.5 ^ab^	12.5	0.047
Serine	28.0 ^a^	29.1 ^a^	0.6 ^b^	12.17	0.004
Threonine	24.0 ^b^	38.1 ^a^	13.2 ^b^	18.93	0.008
Phenylalanine	47.0	46.1	48.6	0.75	0.165
Aspartic acid	32.4 ^ab^	23.7 ^b^	48.4 ^a^	3.96	0.030
Glutamic acid	205.8 ^a^	147.8 ^a^	0 ^b^	17.46	0.003
Asparagine	21.4	16.4	8.6	2.29	0.078
Ornithine	10.7	10.7	11.0	0.62	0.931
Glutamine	12.5 ^a^	10.1 ^a^	0 ^b^	0.87	0.001
Lysine	135.4	133.4	126.6	15.01	0.925
Tyrosine	39.8	34.5	35.0	3.66	0.633
Tryptophan	52.0	39.9	33.9	7.35	0.383

Different superscripts ^(a,b,c)^ within the same row indicate statistically significant differences between groups (control, *T. pedis*, *T. phagedenis*) based on one-way ANOVA and Duncan’s multiple range test (DMRT) (*p* < 0.05).

**Table 3:** Quantitative analysis of fatty acid (FA) and short-chain fatty acid (SCFA) profiles in culture media.

FA (ng/2 µL)	Control	*T. pedis*	*T. phagedenis*	SEM	*p*-Value
cis-9-Hexadecenoic acid (C16:1)	146.9 ^a^	28.8 ^b^	18.6 ^c^	2.12	<0.001
Palmitic acid (C16:0)	1115.3 ^a^	191.4 ^b^	142.2 ^b^	79.98	<0.001
Linoleic acid (C18:2)	135.1 ^a^	18.5 ^c^	70.5 ^b^	2.29	<0.001
Oleic acid (C18:1)	745.1 ^a^	431.4 ^b^	386.2 ^b^	14.24	<0.001
Octadecanoic acid (C18:0)	730.9	405.7	228.4	159.48	0.203
Octacosanoic acid (C28:0)	257.0	93.4	310.7	100.56	0.523
SCFA (mM)					
Lactic acid	26.1 ^a^	15.28 ^b^	12.99 ^b^	2.15	0.002
Formic acid	0 ^b^	0 ^b^	5.1 ^a^	0.85	<0.001
Acetic acid	22.08 ^b^	50.96 ^a^	26.59 ^b^	4.72	<0.001
Propionic acid	5.73 ^c^	59.18 ^a^	28.41 ^b^	7.79	<0.001
Butyric acid	16.19 ^c^	67.55 ^b^	116.58 ^a^	14.50	<0.001

Different superscripts ^(a,b,c)^ within the same row indicate statistically significant differences between groups (control, *T. pedis*, *T. phagedenis*) based on one-way ANOVA and Duncan’s multiple range test (DMRT) (*p* < 0.05).

## Data Availability

All data included in this study are presented within the manuscript.
